# Tuning the Properties of Thin-Film TaRu for Hydrogen-Sensing Applications

**DOI:** 10.1021/acsami.2c20112

**Published:** 2023-02-03

**Authors:** Lars J. Bannenberg, Herman Schreuders, Nathan van Beugen, Christy Kinane, Stephen Hall, Bernard Dam

**Affiliations:** † Faculty of Applied Sciences, Delft University of Technology, Mekelweg 15, 2629 JBDelft, The Netherlands; ‡ ISIS Neutron Source, Rutherford Appleton Laboratory, STFC, UKRI, OX11 0QXDidcot, United Kingdom

**Keywords:** optical hydrogen sensing, metal hydrides, thin films, tantalum, ruthenium, X-ray diffraction, neutron reflectometry

## Abstract

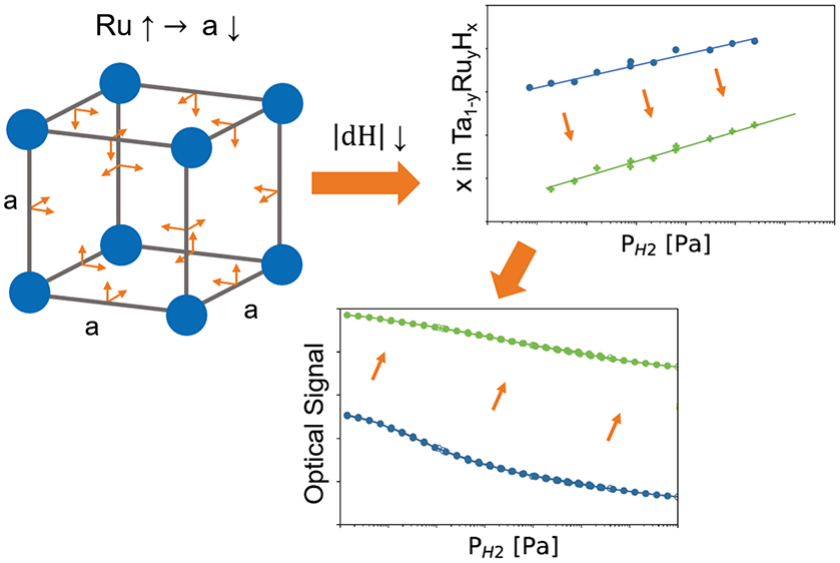

Accurate, cost-efficient, and safe hydrogen sensors will play a key role in the future hydrogen economy. Optical hydrogen sensors based on metal hydrides are attractive owing to their small size and costs and the fact that they are intrinsically safe. These sensors rely on suitable sensing materials, of which the optical properties change when they absorb hydrogen if they are in contact with a hydrogen-containing environment. Here, we illustrate how we can use alloying to tune the properties of hydrogen-sensing materials by considering thin films consisting of tantalum doped with ruthenium. Using a combination of optical transmission measurements, ex situ and in situ X-ray diffraction, and neutron and X-ray reflectometry, we show that introducing Ru in Ta results in a solid solution of Ta and Ru up to at least 30% Ru. The alloying has two major effects: the compression of the unit cell with increasing Ru doping modifies the enthalpy of hydrogenation and thereby shifts the pressure window in which the material absorbs hydrogen to higher hydrogen concentrations, and it reduces the amount of hydrogen absorbed by the material. This allows one to tune the pressure/concentration window of the sensor and its sensitivity and makes Ta_1–*y*_Ru_*y*_ an ideal hysteresis-free tunable hydrogen-sensing material with a sensing range of >7 orders of magnitude in pressure. In a more general perspective, these results demonstrate that one can rationally tune the properties of metal hydride optical hydrogen-sensing layers by appropriate alloying.

## 1 Introduction

Hydrogen is playing a paramount role in the transition to a green economy.^1−4^ Used as a feedstock for the (chemical) industry, large-scale long-term energy storage, and heavy transportation, hydrogen sensors for leak detection are crucial for safety application as well as essential for the reliable and efficient operation of CO_2_ conversion devices and fuel cells.^5,6^ Current commercially available hydrogen sensors, typically relying on catalytic or thermal conductive processes, have only a modest sensing range and are relatively large and costly. Opposed to this, optical hydrogen sensors are relatively small and cheap and feature an enormous sensing range of up to 7 orders in terms of concentration/partial hydrogen pressure.^7−13^ Most importantly, they do not require any electrical currents near the sensing area, making them intrinsically safe. The working principle behind such a sensor is straightforward: when a metal hydride sensing layer is in contact with an environment where hydrogen is present, the metal hydride (partly) hydrogenates, inducing a change in the optical properties of the material. In turn, from measuring, e.g., changes of the optical transmission or reflectivity, one can thus determine the hydrogen pressure/concentration.

The performances of hydrogen sensors are directly related to the intrinsic material properties of hydrogen-sensing materials.^9^ To achieve a large and hysteresis-free sensing range, one should find metal hydrides that gradually absorb hydrogen upon increasing partial hydrogen pressure/concentration without undergoing a phase transition upon hydrogen absorption (i.e., a large solid solution range of hydrogen and the host metal). Plastic deformation, another source of hysteresis, should also be minimized. A high sensitivity can be achieved by materials that absorb vast amounts of hydrogen and where large changes of the optical properties are induced when hydrogen is absorbed. Differently, to achieve short response times, materials with a high hydrogen diffusion coefficient and that absorb small quantities of hydrogen are optimal, while a stable hydrogen sensor can be achieved by selecting a material that only mildly expands upon hydrogen absorption, does not undergo any phase transition upon hydrogenation, and consists of a single phase.

Traditionally, palladium and alloys thereof have been considered for hydrogen sensing. While palladium can dissociate molecular hydrogen and has a modest sensing range at room temperature of 3 orders of magnitude, the alloying of palladium with elements such as Au is required to reduce/eliminate the hysteresis arising from a first-order phase transition upon hydrogen sorption, which drastically lowers the optical response and thus the sensitivity of the sensor.^14−21^ As an alternative, hafnium and especially tantalum-based hydrogen sensors have been developed.^22−25^ These materials are then combined with a suitable capping layer that promotes hydrogen dissociation.^26^ Thin-film tantalum features a sensing range of >7 orders of magnitude in hydrogen pressure/concentration that is free of any hysteresis and completely within one thermodynamic phase. The large solid solution range of hydrogen and tantalum is a pronounced effect of the nanoconfinement of tantalum because in bulk tantalum a series of phase transitions are observed when it absorbs hydrogen.^27^ In fact, even below the ppb regime tantalum already absorbs significant amounts of hydrogen (≈TaH_0.5_), which is wasted because it is not relevant in most applications. This hydrogen needs to dissociate and diffuse before it is accommodated in the lattice, leading to a larger volumetric expansion and slower kinetics than absolutely needed for the pressure range required. Therefore, it would be beneficial to shift the pressure range of tantalum to higher concentrations while preserving the highly sensitive, hysteresis-free, and large sensing range of tantalum.

The purpose of this paper is to illustrate how alloying can be used to rationally tune the sensing properties of metal hydride sensing materials by assessing the effect of alloying tantalum (body-centered cubic (bcc)) by ruthenium (face-centered cubic (fcc)) to tailor the properties of tantalum (bcc). Alloying with ruthenium is expected to be advantageous because tantalum and ruthenium have a large solid solution range of >20% Ru,^28,29^ implying that relatively large quantities of Ru can be introduced without a phase change/separation occurring that would likely lead to hysteresis or reduced stability. Because ruthenium has a substantially smaller volume per atom (0.0138 nm^3^) than tantalum (0.0181 nm^3^), its alloying will compress the unit cell substantially. In turn, this leads to compressive strain that makes the enthalpy of hydrogenation less favorable and thus reduces the amount of hydrogen absorbed by the layer at low hydrogen pressures.^30^ Substitution of Ta by Ru thus provides more possibilities to tune the pressure range of the sensor than the previously considered substitution by Pd because the volume per atom of palladium (0.0147 nm^3^) is closer to that of tantalum. On top of that, the solid solution window of ∼12% for Pd is much smaller. Higher Pd concentration alloys result in phase separation and a strongly hysteretic response to hydrogen.^24^

The experiments on Ta_1–*y*_Ru_*y*_ thin films demonstrate the positive effect of alloying with Ru. They show that even for *y* = 0.3 a solid solution of Ru and Ta is formed in the body-centered cubic phase of tantalum, while at the same time a significant reduction of the size of the unit cell is obtained. Detailed in situ X-ray and neutron reflectometry measurements indicate that Ru doping is effective in reducing the hydrogen content and the expansion of the lattice. Optical measurements confirm that the sensing range shifts to higher pressures and that the magnitude of the optical changes decreases until for *y* = 0.3 no optical response can be discerned. However, for *y* ≲ 0.12, the sensitivity, being the slope of the changes in optical transmission with changing hydrogen pressure/concentration, is only marginally affected and in some cases even improved. Furthermore, a comparison of the optical response and the amount of hydrogen absorbed by the layer shows that Ru doping reduces the optical changes of the system as induced by the hydrogen absorption of the layer. Taken together, these results demonstrate that one can rationally tune the properties of metal hydride optical hydrogen-sensing layers by appropriate alloying.

## 2 Experimental Section

### 2.1 Sample Fabrication

The Ta_1–*y*_Ru_*y*_ thin-film samples are produced by magnetron sputtering and consist of a 4 nm titanium adhesion layer, a 40 nm Ta_1–*y*_Ru_*y*_ sensing layer, and a 10 nm capping layer to catalyze the hydrogen dissociation and recombination reaction and prevent the film from oxidation (nominal thicknesses). As a capping layer, we have used Pd_0.6_Au_0.4_ for the optical measurements and Pd_0.6_Au_0.35_Cu_0.05_ for the structural measurements. The slight difference in material was for practical reasons. We have no indications that this slight difference in capping materials affects any of the conclusions regarding the Ta_1–*y*_Ru_*y*_ layer. Both materials (i) show hardly any optical response as the material hardly absorbs hydrogen, (ii) do not show a (first-order) phase transition on hydrogen absorption, and (iii) enable a fast response to changing hydrogen pressures. As such, the contribution to the optical signal is small. The reader is kindly referred to our previous work for a more detailed discussion and study on capping layers for metal hydride optical hydrogen sensors.^26^ We note that, to avoid cross-contamination to other chemical species, such as NO_*x*_, CO_2_, CH_4_, CO, and H_2_O, the capping layer can be coated with polymers such as poly(methyl methacrylate) (PMMA) and polytetrafluoroethylene (PTFE).^19,31,32^

All layers were deposited in 0.3 Pa of Ar by magnetron sputtering in an ultrahigh vacuum chamber (AJA Int.) with a base pressure of 10^–6^ Pa. For the optical and X-ray measurements, 10 × 10 mm^2^ quartz substrates were used with a thickness of 0.5 mm and surface roughness <0.4 nm (Mateck GmbH, Jülich, Germany). For the neutron reflectometry measurements, fused quartz substrates with a diameter of 76.2 mm (3 in.) with a thickness of 3.0 mm, a surface roughness <0.5 nm, and a flatness of 2 lambda over 85% CA Central (Coresix Precision Glass, Inc., VA, U.S.A.).

The substrates were rotated to enhance the homogeneity of the deposited layers. Typical deposition rates include 0.10 nm s^–1^ (125 W dc) for Ta, 0.12 nm s^–1^ (100 W dc) for Ru, 0.05 nm s^–1^ (100 W dc) for Ti, 0.13 nm s^–1^ (50 W dc) for Pd, 0.11 nm s^–1^ (25 W dc) for Au (see Table S1 for the exact conditions for each composition), and 0.13 nm s^–1^ (50 W dc) for the custom-made Pd_0.6_Au_0.35_Cu_0.05_ alloy target. All targets have a diameter of 5.08 mm (2 in.) and a purity of at least 99.9% (Mateck GmbH, Jülich, Germany). The deposition rates were determined by sputtering each target independently at a fixed power over a well-defined time interval. Subsequently, X-ray reflectometry (XRR) was used to obtain the layer thickness of this reference sample, on the basis of which we computed the sputter rate. The Ta target was presputtered for 120 min to avoid possible contamination from the tantalum oxide and nitride layers present at the surface of the target. We wish to emphasize that for large-scale manufacturing alloy targets could be used.

The thickness and crystal structure of all samples were verified with X-ray diffraction (XRD) and XRR (Figure S1 and Table S2). The fits to the XRR data (see below for experimental details) reveal that the deviation of the layer thickness between the different samples is at maximum 3%, the density of the various layers is consistent within 3% to the literature value for the bulk material, and the root-mean-square roughness of the various layers is at maximum 1.5 nm. Atomic force microscopy (AFM) measurements (Figure S2) performed on a selection of samples with a Bruker Multimode AFM in tapping mode were used to characterize the morphology of the sample after exposure to hydrogen, confirm the smooth surface, and indicate the absence of mesopores or holes in the film. The root-mean-square roughness of ∼1 nm is in good agreement with the value obtained from X-ray reflectometry (XRR) measurements.

**Figure 1:**
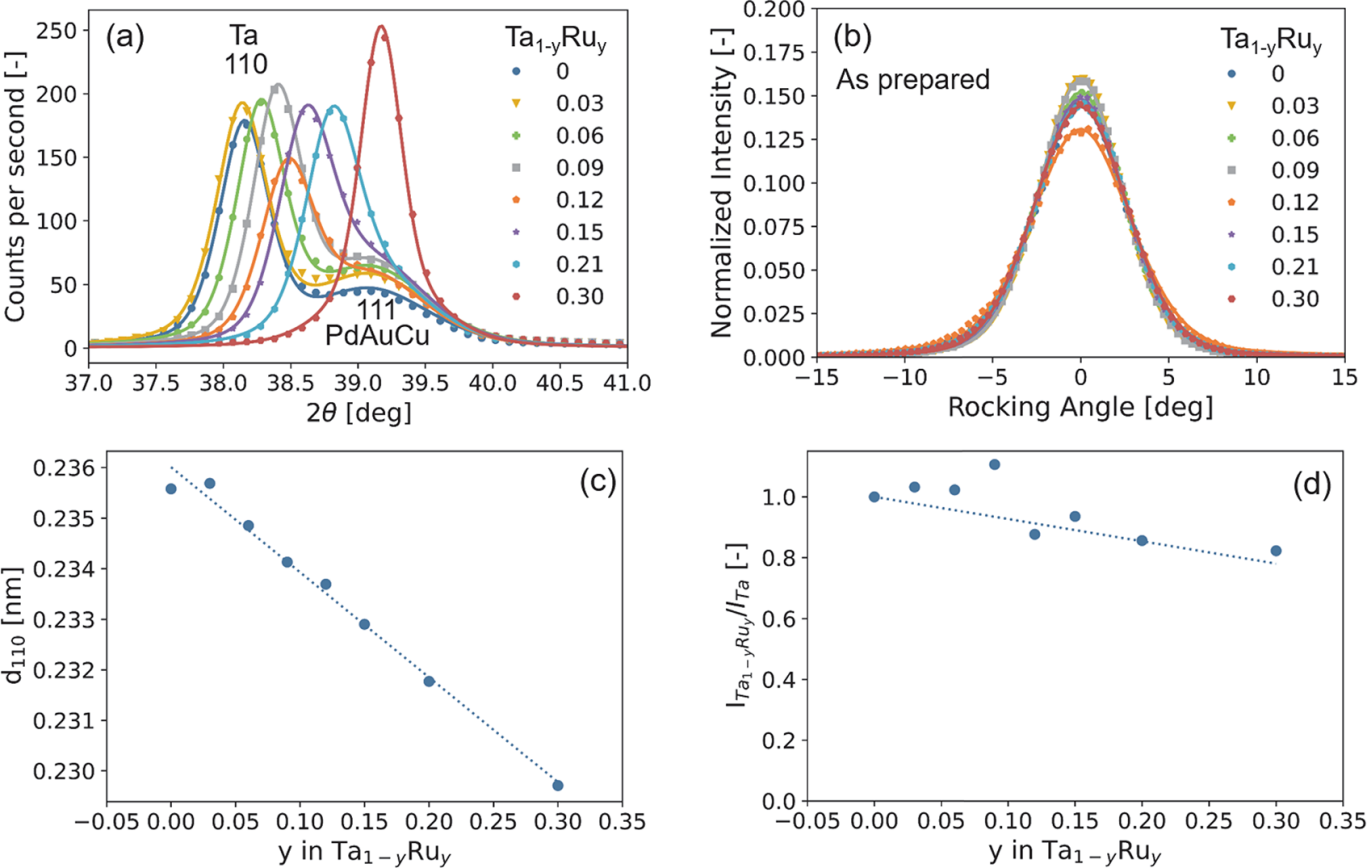
Ex situ X-ray diffraction (XRD) results of the 40 nm Ta_1–*y*_Ru_*y*_ thin films with a 4 nm Ti adhesion layer and capped with a 10 nm Pd_0.6_Au_0.35_Cu_0_.05 layer before exposure of the thin films to hydrogen and measured in air. (a) Diffraction patterns (Cu Kα, λ = 0.1542 nm) of the Ta_1–*y*_Ru_*y*_ thin films. The continuous lines represent fits of two pseudo-Voigt functions to the experimental data accounting for the bcc ⟨110⟩ Ta_1–*y*_Ru_*y*_ and fcc ⟨111⟩ Pd_0.6_Au_0.35_Cu_0.05_ peaks. (b) Rocking curves of the Ta_1–*y*_Ru_*y*_ thin films around the bcc Ta_1–*y*_Ru_*y*_ ⟨110 ⟩ peak. (c) Ru doping dependence of the *d*_110_-spacing in Ta_1–*y*_Ru_*y*_. The continuous line indicates an oridinary least-square fit to the data. (d) Ru concentration dependence of the total intensity of the ⟨110⟩ diffraction peak in Ta_1–*y*_Ru_*y*_, in which the effect of both the changing amplitude and width are incorporated. It is computed by multiplying the integrated intensity of the fitted ⟨110⟩ Ta_1–*y*_Ru_*y*_ peak by the full width at half maximum (fwhm) of the rocking curve of (b). The intensity is subsequently scaled to the intensity of the Ta sample. The dashed line indicates the theoretically expected Ru-concentration dependence of the intensity according to eq 2.

Prior to all measurements, the thin films were exposed to three cycles of hydrogen with a maximum pressure of *P*_H2_ = 10^+6^ Pa at *T* = 28 °C. Reproducible and hysteresis-free results were obtained from the second cycle onward. Such deviations between the first and subsequent cycles are usual for thin-film metal hydrides because, in general, a few cycles of exposure to hydrogen are required to show reproducible results due to a settling of the microstructure. Indeed, we find evidence of substantial rearrangements within the films as the *d*-spacing of the Ta_1–*y*_Ru_*y*_ layer decreases (Figures 1a and S3a) and the preferred orientation improves (Figures 1b and S3b) after exposure to hydrogen, especially for compounds with *y* ≲ 0.15.

### 2.2 Structural Measurements

Ex situ and in situ X-ray diffraction (XRD) and X-ray reflectometry (XRR) measurements were performed with a Bruker D8 Discover (Cu Kα, λ = 0.1542 nm) equipped with a LYNXEYE XE detector. The ex situ XRD measurements were performed with a constant footprint of 3 mm achieved using a variable slit on both the primary and secondary sides with the detector operated in 1D mode. The in situ XRD measurements were performed with a Göbel mirror and a 0.2 mm fixed slit on the primary side and two 0.2 mm slits on the secondary side with the detector operated in 0D mode.

The ex situ XRR measurements were performed with a Göbel mirror and a 0.1 mm fixed slit on the primary side and two 0.1 mm slits on the secondary side with the detector operated in 0D mode, while 0.2 mm fixed slits were used for the in situ XRR measurements. The data were fitted with GenX3^33^ to obtain estimates for the layer thickness, roughness, and density of the thin films.

The in situ XRD and XRR measurements were performed inside an Anton Paar XRK900 Reactor chamber with a base pressure of *P* < 5 × 10^–4^ mbar that is connected to an Anton Paar TCU 700 control unit (Anton Paar GmbH, Graz, Austria). A pressure-control unit (MKS Inst., Inc., type 250 controller, Andover, MA, U.S.A.) is connected to a solenoid inlet valve (MKS Inst. 0248AC-10000SV) and a manometer (MKS Inst., Inc., Baratron type 627DMCC1B) that are connected to the inlet of the reactor chamber. The outlet of the reactor chamber is connected to a mass flow controller (Brooks Instruments 150 sccm, Hatfield, PA, U.S.A.) and subsequently a vacuum pump (Adixen Drytel 1025, Pfeiffer Vacuum GmbH, Asslar, Germany). To ensure sufficient flow at low absolute pressures, a solenoid outlet valve (MKS Inst., Inc., 0248AC-10000SV, Andover, MA, connected to a Delta Elektronica ES030-5 Power Supply, Zierikzee, The Netherlands) is positioned parallel to the flow controller. The pressure-control unit, outlet valve, and flow controller are controlled by a home-written National Instruments Labview code.

Neutron reflectometry measurements were performed both at the time-of-flight neutron reflectometer ROG located at the 2.3 MW HOR reactor of the Delft University of Technology, Delft, The Netherlands, and at Offspec, located at the ISIS pulsed neutron source, Didcot, United Kingdom.^34^ At the ROG, the double disc chopper was set to a frequency of 17.7 Hz with an interdisc distance of 0.280 m resulting in a wavelength resolution of Δλ λ ≈ 2.5%. The incident angle was set to 8.5 mrad, with the spectrum between 0.11 < λ < 1.0 nm leading to a *Q*-range of 0.11 < *Q* < 0.98 nm^–1^. A first slit of 1.5 mm and a second slit of 0.75 mm were used, of which the latter one was positioned ∼150 mm from the sample, resulting in a footprint of 80 × 40 mm^2^ (umbra/penumbra) and a resolution of Δ*Q*/*Q* ≈ 0.05%. The neutrons were detected using a ^3^He detector. The measurements at Offspec were performed with an incident angle of 11 mrad, leading to 0.1 < *Q* < 0.84 nm^–1^. The slits were set to *D*_1_ = 4.184 mm and *D*_2_ = 0.5025 mm, providing a footprint of 44/75 × 30 mm^2^ umbra/penumbra and a resolution of Δ*Q*/*Q* ≈ 0.04%. The neutrons were detected using a position-sensitive detector.

The samples were hydrogenated inside a temperature- and pressure-controlled cell as described elsewhere^35^, and this was performed at *T* = 22 °C. The partial hydrogen pressure was varied by controlling the absolute pressure of 0.1% or 4.0% H_2_ in Ar gas between 1.5 mbar and 6.1 bar.

The data were fitted with GenX3^33,36^ to obtain estimates for the layer thickness, roughness, and scattering length density (SLD) for all three layers. In the analysis of the XRR results, we fixed the thickness of the Ti and Pd_0.6_Au_0.35_Cu_0.05_ film to the value when analyzing the XRR results owing to a high correlation between the thickness of the three layers because our primary interest is in the thickness of the Ta_1–*y*_Ru_*y*_ layer. As the 10 nm Pd_0.6_Au_0.35_Cu_0.05_ also expands, this results in a slight overestimation of the expansion of the Ta_1–*y*_Ru_*y*_ layer. Subsequently, from the fitted SLDs and thicknesses of the layers, we can compute the hydrogen concentration of the layer using
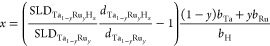
1where SLD = ∑_*i*=1_^*N*^*b*_*iN_i_*_ is the SLD of the layer; *b*_Ta_ = 6.91 fm, *b*_Ru_ = 7.03 fm, and *b*_H_ = −3.739 fm are the scattering lengths of tantalum, ruthenium, and hydrogen, respectively;^37^ and *N*_*i*_ is the number of atoms *i* per volume unit.^38^

### 2.3 Optical Measurements

The optical transmission was measured with hydrogenography^39^ with an Imaging Source DFK 23UM021 1/3 in. Aptina CMOS MT9M021 color camera 1280 × 960 pixel color camera with an Edmunds Optics 55-906 lens, i.e., the same lens as used in ref (38), and five Philips MR16 MASTER LEDs (10/50 W) with a color temperature of 4 000 K as a light source (Figure S4). The camera records the red, green, and blue domains separately, and the wavelength-dependent sensitivities of each color channel are displayed in Figure S5. The transmission is averaged over an area of ∼80 mm^2^ corresponding to roughly 100 × 100 pixels. A reference sample is used to compensate for fluctuations of the LED white light source and correct for the response of the 10 nm Pd_0.60_Au_0.40_ capping layer. The partial hydrogen pressures of 10^–1^ < *P*_H2_ < 10^+6^ Pa are obtained by using 0.10%, 4.0%, and 100% H_2_ in Ar gas mixtures (Δ*c*_H2_/*c*_H2_ < 2%, Linde Gas Benelux BV, Dieren, The Netherlands) and varying the total pressure inside the chamber between 0.15 < *P*_tot_ < 1.0 × 10^+6^ Pa. Typical gas flows are 20 sccm for increasing pressure steps and 100 sccm for decreasing pressure steps.

## 3 Results

### 3.1 Structural Behavior

#### 3.1.1 Ex Situ X-ray Diffraction

To assess whether substitution of Ta by Ru is beneficial for hydrogen-sensing applications, we first investigate whether a solid solution is formed between Ta and Ru. This is important as a solid solution of Ta in Ru in the Ta bcc phase would likely retain the favorable hydrogen-sensing properties of tantalum, while for example a material that is phase-separated may result in hysteresis and a compromised long-term instability as the phase fractions may vary over time.

Similar to bulk Ta_1–*y*_Ru_*y*_,^29^ the X-ray diffraction (XRD) measurements of Figure 1 reveal that the Ta_1–*y*_Ru_*y*_-based thin films form a solid solution with no signs of phase segregation for all compounds considered. For all of the as-prepared films, only the ⟨110⟩ bcc Ta diffraction peak is observed, indicating that the films are strongly textured with the ⟨110⟩ in the out-of-plane direction. On the basis of the fwhm β of the XRD peak and Scherrer’s equation , with *K* = 0.9 being the dimensionless shape factor and λ = 0.154 nm the wavelength of the X-rays, we estimate that the average crystallite size is about τ = 2 × 10^2^ nm, which is more or less constant with composition. Most importantly, consistent with the formation of a solid solution, we observe an almost linear decrease of the *d*_110_-spacing with increasing Ru concentration (Figure 1c).

Furthermore, to exclude the possibility that other phases are formed that cannot be directly detected by XRD, we consider the total intensity of the diffraction peak. This analysis is complicated by the strong texture of the thin films and potential differences in preferred orientation between the samples, for which we adjust by taking into account the width of the rocking scan of Figure 1b. Theoretically, for a solid solution of Ta and Ru, we expect a decreasing intensity, as a replacement of Ta (*Z* = 73) by Ru (*Z* = 44) reduces for a bcc structure the fraction of X-rays diffracted by the material according to
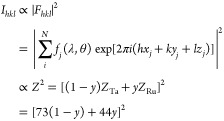
2where *F*_*hkl*_ is the structure factor, *N* and *x*_*j*_, *y*_*j*_, and *z*_*j*_ are the number and relative coordinates of the atoms in the primitive (bcc) unit cell, and *f*_*j*_(λ, θ) is the form factor that can be approximated as *f*_*j*_(λ, θ) ≈ *Zr*_e_*g*(θ, λ). If additional phases are formed, we would observe a much stronger decrease of the total intensity than expected based on eq 2 because amorphous parts of the material no longer contribute to the intensity of the diffraction peak. However, the results show a linear decrease of the total intensity of the diffraction peak that is close to what is expected based on eq 2. This result is thus consistent with the fact that no other (amorphous) phases are present and a solid solution of Ta and Ru is formed.

Second, the ex situ XRD results show that Ru substitution is successful in compressing the Ta unit cell. Figure 1c shows that the *d*_110_-spacing is reduced from 0.236 nm for Ta to 0.230 nm at Ta_0.7_Ru_0.3_, which is equivalent to a substantial 7.4% decrease in unit cell volume. This implies that Ru doping is a successful method to compress the Ta unit cell and more successful than substitution by Pd. Indeed, for Ta_1–*y*_Pd_*y*_ a solid solution range is present up to *y* ≈ 12%, at which point the unit cell is compressed by 1.5%.^24^ This is important because a larger unit cell compression results in a larger shift of the equilibrium pressure of the metal hydride and thus induces a bigger shift of the hydrogen-sensing range of the material. As such, Ru substitution allows for more possibilities and flexibility to tailor the properties of the materials.

#### 3.1.2 In Situ X-ray Diffraction

Another important property of hydrogen-sensing materials is that they should not possess any (first-order) phase transitions when exposed to hydrogen. Phase transitions result in a hysteretic response, longer response times, and a potentially compromised stability of the material upon repeated exposure to hydrogen. Furthermore, the material should not plastically deform when it expands to accommodate the hydrogen because such a plastic deformation is an additional source of hysteresis.^20,40,41^

**Figure 2:**
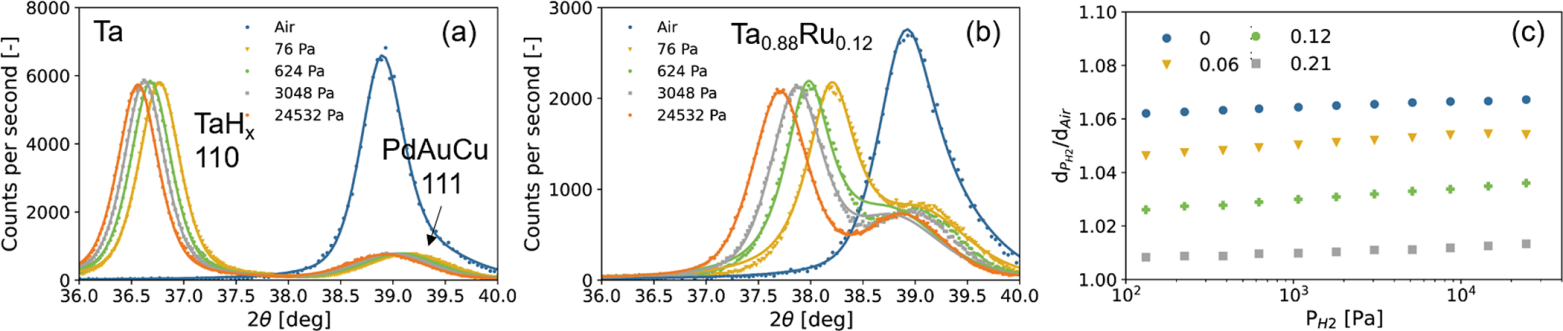
In situ XRD results of the 40 nm Ta_1–*y*_Ru_*y*_ thin films with a 4 nm Ti adhesion layer and capped with a 10 nm Pd_0.6_Au_0.35_Cu_0.05_ at *T* = 25 °C. (a, b) Diffraction patterns (Cu Kα, λ = 0.154 nm) of the Ta_1–*y*_Ru_*y*_ thin films with (a) *y* = 0 and (b) *y* = 0.12 measured for the hydrogen pressures indicated in the legend and for decreasing pressure steps. The continuous lines represent fits of two pseudo-Voigt functions to the experimental data. (c) Hydrogen pressure dependence of the expansion of the *d*_110_-spacing in Ta_1–*y*_Ru_*y*_ relative to the unloaded state in air.

We investigate the presence of phase transitions by in situ XRD, for which the results are presented in Figure 2. In these measurements, we expose our thin film to stepwise changing hydrogen partial pressures and measure the 110 diffraction peak. At the same time, we also perform in situ XRR measurements to probe the thickness of the film. We note that the air state refers to the measurement after we have exposed the film to hydrogen. With respect to the measurements of Figure 1a, this peak is slightly shifted, indicating a more compact structure owing to a settlement of the microstructure of the material upon first exposure to hydrogen. Such effects are common to metal hydrides and are seen, for example, in Pd and Pd-based materials.^23,40,41^

With increasing hydrogen pressure and for all compounds studied, the diffraction peak continuously shifts to lower angles (Figure 2a and b) without any change in its peak shape or the appearance of an additional peak. As such, no hint of any (first-order) phase transition is detected. The absence of first-order phase transitions thus paves the way for a hysteresis-free optical response of the hydrogen-sensing material.

**Figure 3:**
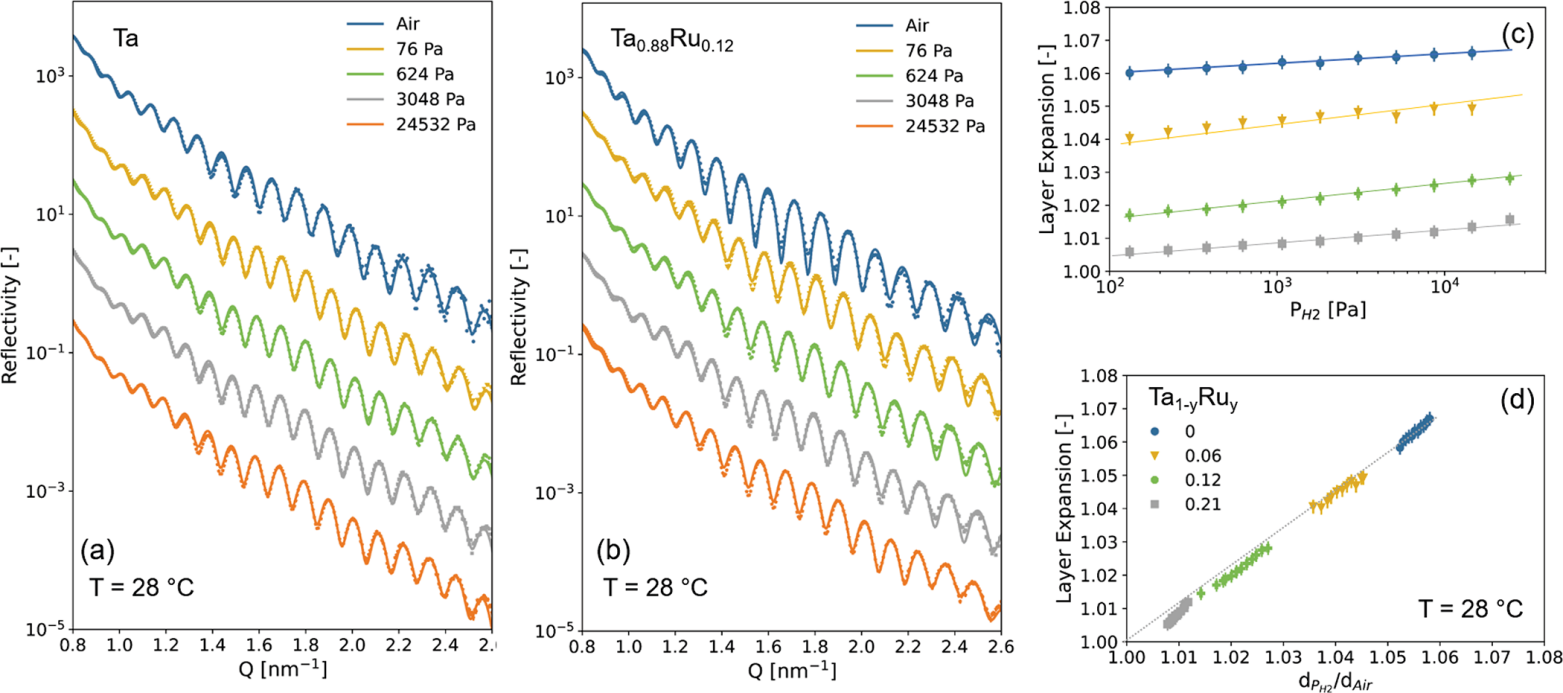
In situ XRR results of the 40 nm Ta_1–*y*_Ru_*y*_ thin films with a 4 nm Ti adhesion layer and capped with a 10 nm Pd_0.6_Au_0.35_Cu_0.05_ at *T* = 25 °C. (a, b) Reflectograms of the Ta_1–*y*_Ru_*y*_ thin films with (a) *y* = 0 and (b) *y* = 0.12 measured for the hydrogen pressures indicated in the legend and for decreasing pressure steps. The continuous lines represent fits of a model to the data on the basis of which estimates for the density and layer thickness are obtained (see Figure S6 for the scattering length density profiles). (c) Hydrogen pressure dependence of the expansion of the layer thickness of Ta_1–*y*_Ru_*y*_ relative to the unloaded state in air. (d) Relation between the *d*_110_-spacing and the layer thickness. The line indicates an ordinary least-squares fit to the data.

Furthermore, the in situ XRD results of Figure 2 indicate a gradual hydrogenation and expansion of the Ta_1–*y*_Ru_*y*_H_*x*_ bcc unit cell on hydrogenation. This is underscored by the complementary in situ XRR measurements of Figure 3, which show a gradual expansion of the film’s layer thickness. Importantly, both the XRD and XRR measurements indicate that the volumetric expansion is substantially reduced with Ru substitution (Figure 3c; see Figure S6 for the corresponding scattering length density profiles). While an expansion of ∼6% for Ta is found at *P*_H2_ = 10^+4^ Pa (10% of H_2_ in air), for Ta_0.88_Ru_0.12_ this is reduced to 3%. The reduced expansion of the unit cell likely correlates with a reduced hydrogen content (which is evaluated in the next section). For practical purposes, a reduced expansion makes the thin films less prone to cracking and delamination. Furthermore, we find that, for the expansion within the pressure window most interesting to leak detection for safety applications, i.e., 10^+2^ < *P*_H2_ < 10^+4^ Pa, the increase in layer expansion is larger for the Ru-doped compounds and ∼50% times larger for Ta_0.88_Ru_0.12_. This can be beneficial for hydrogen sensors that make use of the volumetric expansion such as fiber Bragg gratings,^7^ as here the larger expansion results in a larger sensitivity/resolution with which the partial hydrogen pressure can be determined.

In these alloys as well as in pure Ta, we observe a substantial deformation of the unit cell upon hydrogenation, i.e., becoming slightly tetragonal. The details of this nanoconfinement-induced effect^20,38,42−46^ are discussed for Ta elsewhere.^27^ In short, in bulk the relationship between the volume of a cubic unit cell and the *d*-spacing is *V* ∝ *d*_*hkl*_^3^, and the hydrogen-induced expansion is accommodated in all directions. In the case of the clamped thin films studied here, *V* ∝ *d*_*hkl*_ is observed, and the volumetric expansion is realized by expanding the unit cell in the out-of-plane direction only. As a result, the XRD measurements show the complete absence of any hysteresis upon hydrogenation of the film. This implies that the hydrogenation takes place without any plastic deformation. This is a major benefit of Ta_1–*y*_Ru_*y*_, as other thin-film materials such as Pd_1–*y*_Au_*y*_^20,38,42−46^ plastically deform upon hydrogenation and thereby have a hysteretic response to hydrogen.

#### 3.1.3 In Situ Neutron Reflectometry

As the optical signal upon exposure to hydrogen is induced by the absorption of hydrogen by the sensing material, the amount of hydrogen absorbed is a key parameter determining the sensor performance. Furthermore, the amount of hydrogen atoms absorbed determines the response time, which is mainly limited by the molecular dissociation into atomic hydrogen.^19,26^

**Figure 4:**
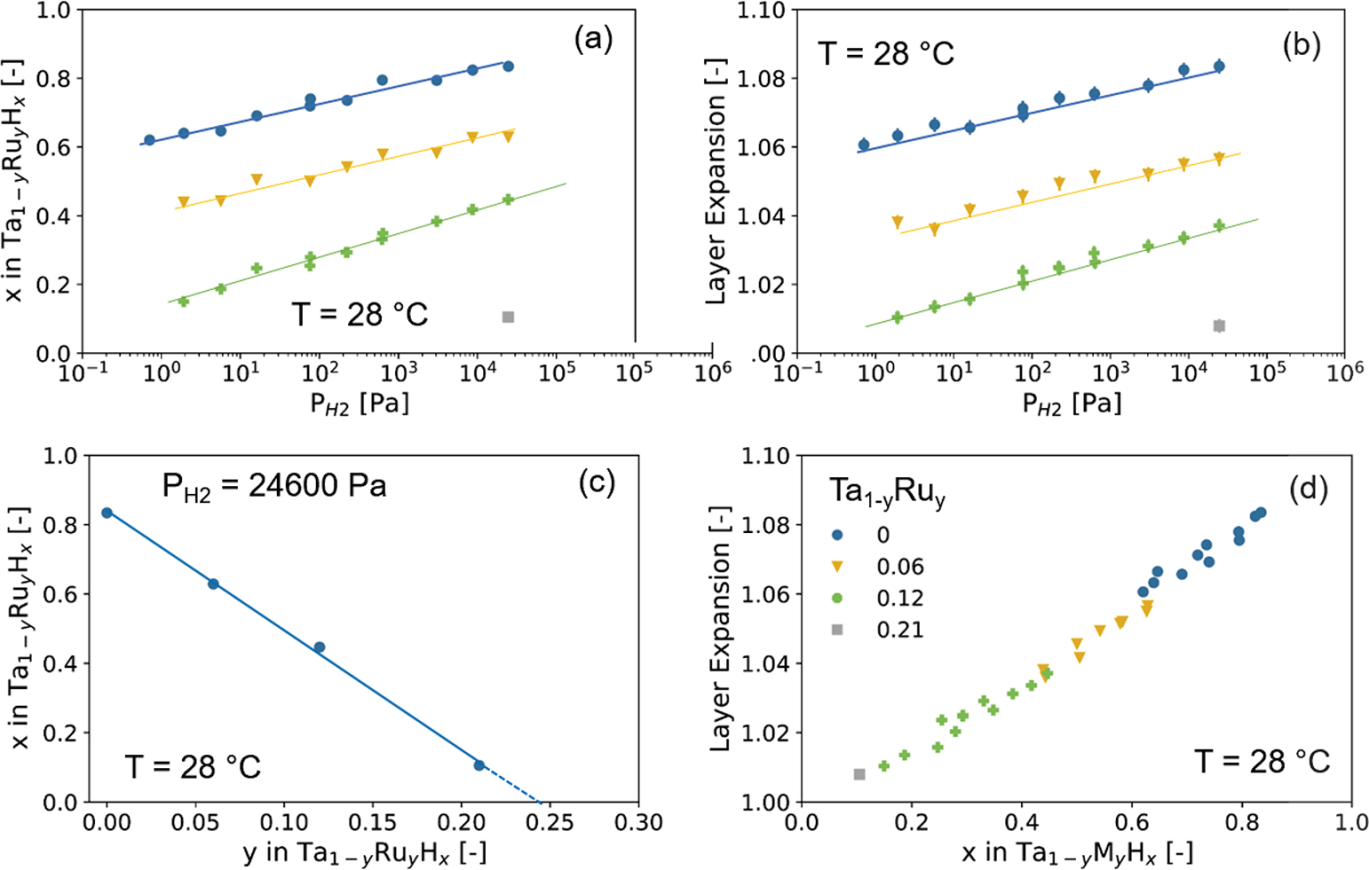
In situ NR results of the 40 nm Ta_1–*y*_Ru_*y*_ thin films with a 4 nm Ti adhesion layer and capped with a 10 nm Pd_0.6_Au_0.35_Cu_0.05_ at *T* = 22 °C. The data with the corresponding fits and SLD profiles can be found in Figure S7. (a) Hydrogen pressure dependence of the amount of hydrogen absorbed by the Ta_1–*y*_Ru_*y*_H_*x*_ layer. (b) Hydrogen pressure dependence of the expansion of the Ta_1–*y*_Ru_*y*_ layer. (c) Ru concentration dependence of the amount of hydrogen absorbed by the Ta_1–*y*_Ru_*y*_H_*x*_ layer at *P*_H2_ = 25 400 Pa. (d) Relation between the amount of hydrogen absorbed and the expansion of the Ta_1–*y*_Ru_*y*_ layer. The lines serve as guides to the eyes.

Figure 4 presents the amount of hydrogen absorbed and thickness expansion of the Ta_1–*y*_Ru_*y*_ sensing layer as obtained from neutron reflectometry, for which the raw data, alongside the fits, are displayed in Figure S7. Neutron reflectometry shows that Ru substitution is indeed effective in reducing the amount of hydrogen absorbed. For the partial hydrogen pressure regime relevant for many applications of hydrogen sensors of 1 ≤ *P*_H2_ ≤ 40 000 Pa (0.01% ≤ *c*_H_ ≤ 40%) at room temperature, we observe that the hydrogen content decreases with increasing Ru substitution. This becomes most apparent in Figure 4c, where for a given hydrogen pressure of *P*_H2_ = 24 600 Pa a linear decrease of the amount of hydrogen absorbed as a function of Ru doping is observed. Extrapolating this relation would imply that a sample with a Ru concentration of *y* ≈ 0.25 would absorb no hydrogen at this hydrogen pressure. This would thus suggest that no optical contrast is seen for *y* ≳ 0.25.

The sensitivity of a hydrogen sensor, that is, the accuracy with which one can determine the hydrogen pressure, is directly related to the derivative of the hydrogen content of the layer with respect to the partial hydrogen pressure, i.e., the slope in Figure 4a (see later discussion around eq 5). We find that the slope in Figure 4a is substantially larger for Ta_1–*y*_Ru_*y*_ with *y* = 0.12 than for Ta. As such, this would, ceteris paribus, result in a larger sensitivity of the hydrogen-sensing material.

There could be at least two factors underlying the reduction of the amount of hydrogen absorbed. First, the amount of hydrogen absorbed by the layer can be reduced by Ru atoms blocking the interstitial sites where hydrogen can reside in the bcc structure of Ta.^47^ This could be similar to the doping of Pd with alloyants such as Au that itself does not bond with hydrogen and thereby blocks the hydrogenation sites. In the case of tantalum, the interstitial sites occupied by hydrogen are the tetrahedral sites, of which there are 6 × 4 × 0.5 per unit cell (6 faces, 4 sites per face, each face is shared by two unit cells) that consist of 2 Ta atoms, and these sites are located on the faces of the cubic structure. As such, from each Ru atom, there are 6 (sets of) neighboring sites at equal distance.

Another factor that could be at play here is that the reduced amount of hydrogen absorbed is the result of the lattice compression introduced by the substitution of Ta by Ru. Indeed, introducing 30% of Ru compresses the lattice constant by 2.5% and the volume of the unit cell by ∼8% (Figure 1c) owing to the smaller size of the Ru atom. Generally speaking, a smaller lattice of the host metal results in a less favorable enthalpy of hydrogenation. Thus, to achieve a certain hydrogen concentration in the host metal, a higher hydrogen pressure is required if the volume of the host metal is smaller.^30,48^

### 3.2 Optical Response and Sensing Range

The ex situ and in situ structural characterizations of the Ta_1–*y*_Ru_*y*_ thin films show that a solid solution of Ta and Ru is formed for all compounds considered, that with increasing Ru substitution the unit cell is compressed, while upon exposure to hydrogen no first-order phase transitions or plastic deformations occur. Furthermore, the neutron reflectometry results indicate that the Ru substitution reduces the amount of hydrogen absorbed by the alloy. At the same time, we find that  is larger for Ta_1–*y*_Ru_*y*_ with *y* = 0.12 than for Ta. Taken together, this suggests that (i) the optical response of Ta_1–*y*_Ru_*y*_ should be free of any hysteresis, (ii) the absolute magnitude of the optical response should reduce with increasing Ru concentration, and (iii) the sensitivity of the sensing material for *y* = 0.12 is improved with respect to that of Ta for the pressure range 1 ≤ *P*_H2_ ≤ 40 000 Pa (0.01% ≤ *c*_H_ ≤ 40%).

**Figure 5:**
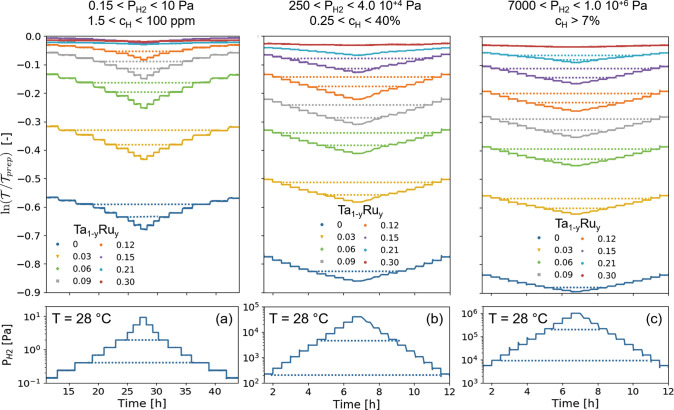
Changes of the green light optical transmission  of the 40 nm Ta_1–*y*_Ru_*y*_ thin film. All samples have a 4 nm Ti adhesion layer capped with a 10 nm Pd_0.60_Au_0.35_Cu_0.05_ layer, the contribution of which was subtracted by subtracting the response of a non-hydrogenating 40 nm Ta_0.5_Pd_0.5_ with the same capping and adhesion layers. The film was exposed at *T* = 28 °C to various increasing and decreasing pressure steps of (a) 1.5 × 10^–1^ ≤ *P*_H2_ ≤ 1.0 × 10^+1^ Pa, (b) 2.5 × 10^+2^ ≤ *P*_H2_ ≤ 4.0 × 10^+4^ Pa, and (c) 7 × 10^+3^ ≤ *P*_H2_ ≤ 1.0 × 10^+6^ Pa. The dashed lines indicate levels of the same transmission (top panel) and pressure (bottom panel). The hydrogen concentrations indicated correspond to an environment with a total pressure of *P*_tot_ = 10^+5^ Pa.

To assess the dependence of the optical properties on the partial hydrogen pressure for thin-film Ta_1–*y*_Ru_*y*_, we measure the changes of the optical transmission  relative to that of the as-prepared state  when applying a series of increasing and decreasing partial hydrogen pressure steps at room temperature. These partial hydrogen pressures are achieved by varying the total pressures of 0.1%, 4%, and 100% of H_2_ in Ar gas inside a temperature-controlled pressure cell. Figure 5 displays the corresponding results for the green-light optical transmissions in three different hydrogen pressure/concentration regions.

For all samples considered and for all three pressure regions, the optical transmission decreases monotonically with increasing pressure and the response is completely free of hysteresis. Indeed, the levels of transmission are well-defined and stable for a given partial hydrogen pressure, and, most importantly, the optical transmission is precisely the same after increasing and decreasing pressure steps. This is in accordance with the in situ XRD results, which reveal that no (first-order) phase transitions or plastic deformations occur when the films are exposed to hydrogen.

**Figure 6:**
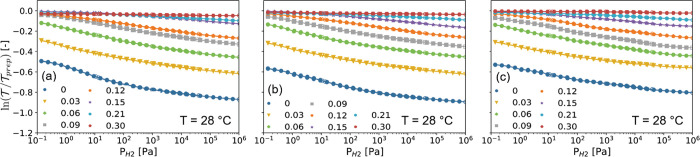
Partial hydrogen pressure dependence of the optical transmission  of 40 nm Ta_1–*y*_Ru_*y*_ sensing layers measured relative to the optical transmission of the as-prepared state () at *T* = 28 °C. Each data point corresponds to the optical transmission after exposing the film for at least 30 min to a constant pressure of *P*_H2_ = 10^–1^–10^+6^ Pa, where the closed data points correspond to increasing pressure steps and the open points correspond to decreasing pressure steps. The data in (a) correspond to the red, in (b) correspond to the green, and in (c) correspond to the blue spectrum (see Figure S5).

**Figure 7:**
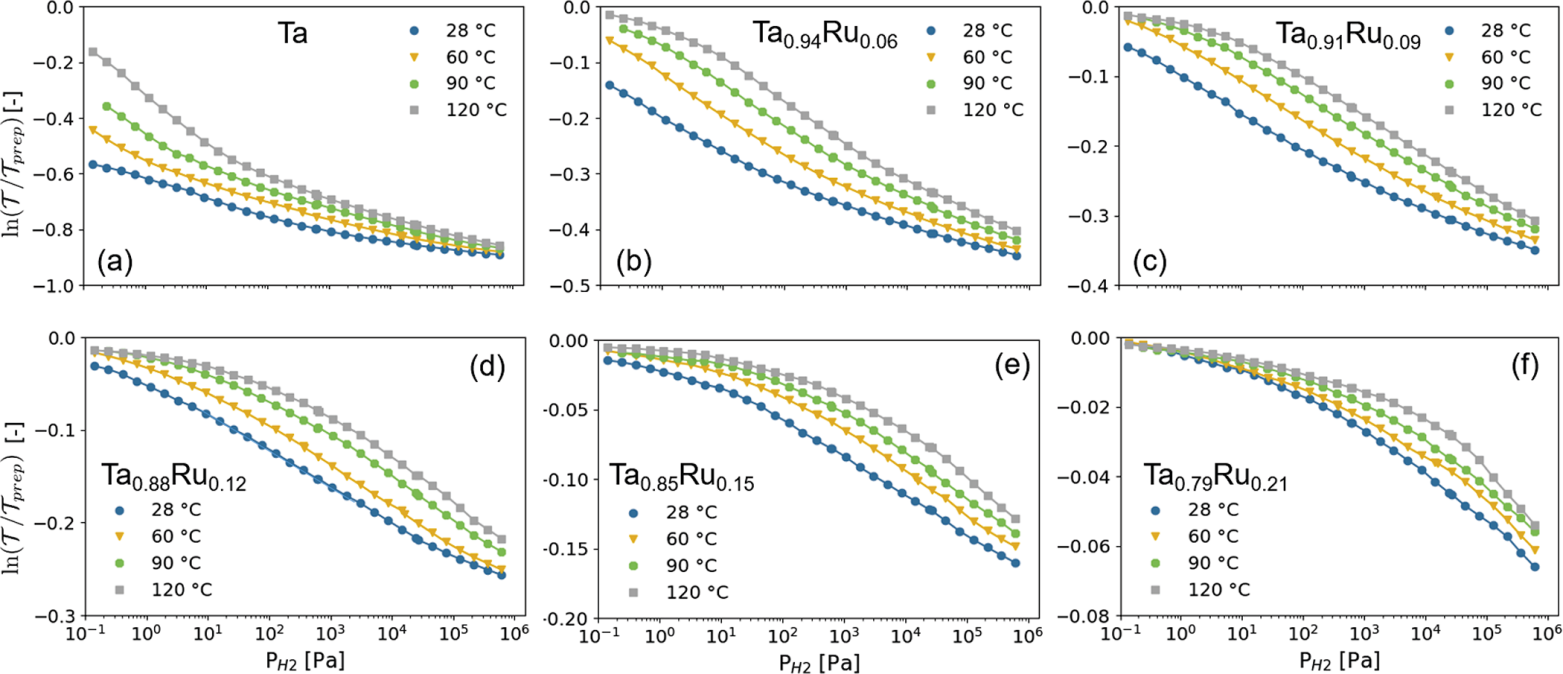
Partial hydrogen pressure and temperature dependence of the optical transmission  measured relative to the optical transmission of the as-prepared state () at *T* = 28 °C of the 40 nm Ta_1–*y*_Ru_*y*_ sensing layers with (a) *y* = 0, (b) *y* = 0.06, (c) *y* = 0.09, (d) *y* = 0.12, (e) *y* = 0.15, and (f) *y* = 0.20. Each data point corresponds to the optical transmission after exposing the film for at least 30 min to a constant pressure of *P*_H2_ = 10^–1^–10^+6^ Pa.

The optical response of the sensing layers is summarized in Figure 6 for a set of different pressures between 1.0 × 10^–1^ < *P*_H2_ < 1.0 × 10^+6^ Pa, for various compositions with 0.0 ≤ *y* ≤ 0.3 at room temperature (data at elevated temperature is available in Figure 7). In this figure, we plot the pressure-transmission isotherms (PTIs) of the sensing layers for three different colors of light, where each closed data point corresponds to the optical transmission obtained after exposing the film for at least 30 min to a constant pressure after an increase in pressure, and the open points correspond to the transmission measured after decreasing the pressure.

The PTIs at room and elevated temperatures reveal for all Ta_1–*y*_Ru_*y*_ films an excellent optical response with no hysteresis and a large and almost constant sensitivity over an extremely wide sensing range. With increasing Ru concentration, the change of the optical response at a given pressure becomes smaller, until the point that for *y* = 0.3 no optical response can be discerned. This is in perfect agreement with the neutron reflectometry results, which indicate that the amount of hydrogen absorbed by the Ta_1–*y*_Ru_*y*_ layer decreases with increasing Ru concentration up to the point where no hydrogen is absorbed (at these pressures) for *y* ≳ 0.25.

Most importantly, we find that Ru substitution is an efficient way to tune the sensing range. The sensing range shifts to higher pressures with increasing Ru substitution. This can be seen, for example, by comparing the PTIs for Ta and Ta_0.85_Ru_0.15_ in Figure 6. Whereas the optical response to hydrogen for Ta starts at a pressure much lower than the lowest pressure measured, i.e., well below 10^–1^ Pa, for Ta_0.85_Ru_0.15_ no sizable response is seen for *P*_H2_ ≲ 10^+1^ Pa. This thus indicates not only that the amount of hydrogen absorbed by the material is reduced but also that the pressure range is successfully shifted to higher hydrogen pressures with increasing Ru doping.

**Figure 8:**
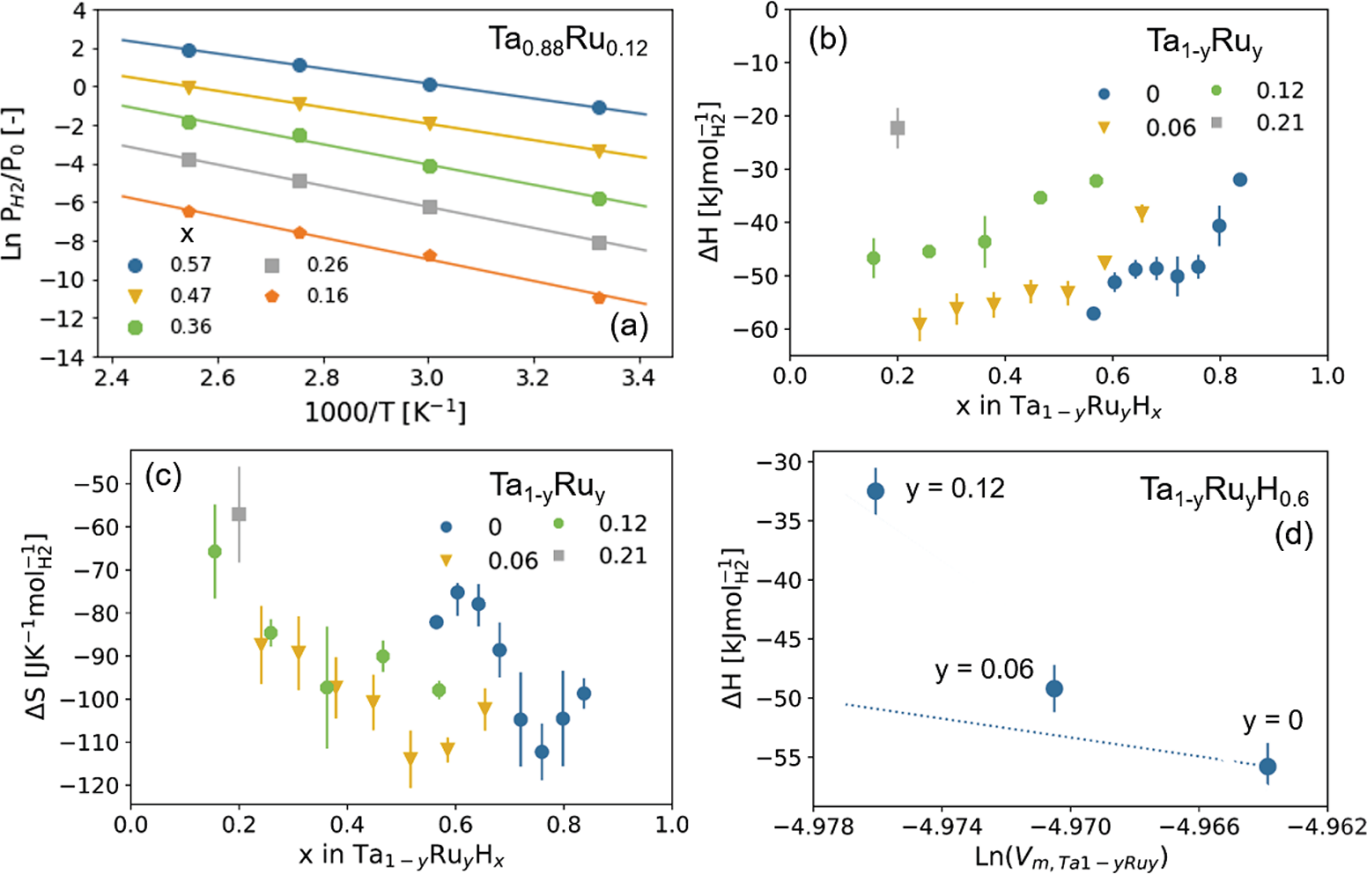
van’t Hoff analysis. (a) Fit of van’t Hoff’s law (eq 3) to the experimental data for Ta_0.88_Ru_0.12_. The analysis is based on the temperature-dependent optical transmission data of Figure 7. The optical transmission is converted to the hydrogen-to-metal ratio that is provided in the figure legend using the scaling obtained in Figure 10. As such, each line corresponds to a fit at a different optical transmission/hydrogen concentration. (b) Enthalpy and (c) entropy of the hydrogenation reaction obtained from similar fits made for four different Ru concentrations. (d) Relation between the enthalpy of hydrogenation of Ta_1–*y*_Ru_*y*_H_0.6_ and the partial volume of the host metal for a single hydrogen concentration. The dashed line indicates the expected trend based on eq 4, the bulk modulus of Ta, and the partial molar volume of hydrogen in Ta.

To further investigate this shift of the pressure window, we determine the enthalpy Δ*H* [kJ mol_H2_^–1^] and entropy Δ*S* [J K mol_H2_^–1^] of the hydrogenation reaction that together dictate the temperature-dependent pressure window of the sensing material. We do this by using van’t Hoff’s law,
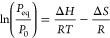
3with *P*_eq_ being the equilibrium hydrogen pressure for a certain hydrogen-to-metal ratio, *P*_0_ = 101.325 kPa being the standard pressure, and *R* = 8.314 J K mol^–1^ being the gas constant.^9^ To estimate the values of Δ*H* and Δ*S* for various hydrogen-to-metal ratios, we consider a set of optical transmission levels. For each of these optical transmission levels, we determine *P*_eq_ for the four temperatures measured (Figure 7) and plot  as a function of the inverse temperature in Figures 8 and S8. Subsequently, we fit these points to eq 3 to obtain the values of Δ*H* and Δ*S* at this optical transmission level. Using the scaling between the optical transmission and the hydrogen-to-metal ratio obtained by neutron reflectometry given in Figure 10c, we can then translate the optical transmission to the hydrogen-to-metal ratio.

The resulting values of Δ*H* and Δ*S* are reported in parts b and c of Figure 8, respectively. For tantalum, the obtained values for the thin films are within the (large) bandwidth reported in the literature. On top of that, similar to the reports in the literature, a nonmonotonic relation between the enthalpy and entropy and the hydrogen-to-metal ratio is obtained (see, e.g., refs (47 and 49)) Most importantly, the results show that, with increasing Ru substitution, the absolute value of the enthalpy of hydrogenation is indeed reduced. This is consistent with the fact that the lattice compression induced by the Ru substitution (Figure 1c) reduces the enthalpy of hydrogenation and thereby shifts the pressure window of the sensing material to higher hydrogen concentrations.

As a next step, we compare the enthalpy of hydrogenation at Ta_1–*y*_Ru_*y*_H_*x*_ to the expected relation based on the lattice compression given by
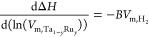
4where *V*_m,Ta_1–*y*_Ru_*y*__ is the molar volume of the host metal, i.e., 1.09 × 10^–5^ m^3^ mol^–1^ for Ta, *B* ≈ 200 GPa is the bulk modulus of Ta, and *V*_m,H_2__ = 2 × 10^–6^ m^3^ mol^–1^ is the partial molar volume of hydrogen in the host lattice (based on the data of Figure 4d).^30^ The results provided in Figure 8d show that the impact of the lattice compression by Ru substitution alone on Δ*H* is insufficient to account for the change in Δ*H*, as the impact is ∼4 times larger than expected based on eq 4. This implies that the change in enthalpy cannot be explained by the lattice compression alone but that other factors such as electronic contributions are also at play. This is consistent with the fact that the optical contrast generation upon hydrogen absorption (discussed in the next section) is affected by the substitution of Ta by Ru (see Figure 10).

### 3.3 Sensitivity, Optical Contrast Generation, and Hydrogen Absorption

The sensitivity of a hydrogen-sensing material is one of its key parameters and defines how accurately the eventual sensor can determine a hydrogen concentration. The sensitivity of a sensing material can therefore be defined as the change of the optical readout parameter, in this case , with respect to the change in hydrogen pressure/concentration. As such, the sensitivity is effectively the slope of the PTIs of Figure 6.

**Figure 9:**
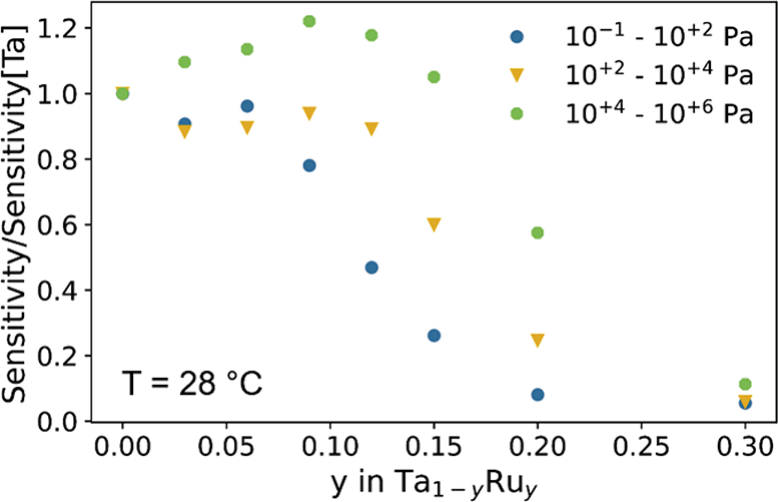
Sensitivity, defined as the average derivative of the optical response  with respect to the partial hydrogen pressure *P*_H2_, computed for the pressure ranges indicated. The sensitivity is normalized to the sensitivity of the Ta sample.

Figure 9 shows that the sensitivity of the material is not reduced by doping with Ru for *y* ≲ 0.12. In fact, for the pressure range 10^+4^ < *P*_H2_ < 10^+6^ (*c*_H2_ > 10%), the sensitivity is increased for all films with 0 < *y* ≲ 0.15 with respect to pure tantalum. On the other hand, for the pressure region of most interest for hydrogen-safety applications, i.e., 10^+2^ < *P*_H2_ < 10^+4^ (0.1 < *c*_H2_ < 10%), no increase in sensitivity is seen, and it is similar for all compounds with *y* ≲ 0.12.

One can go beyond this point and decompose the sensitivity in its different contributions, namely, the change in the amount of hydrogen absorbed by the sensing material  and the magnitude by which the probed optical property changes in the material for every hydrogen atom absorbed, i.e., in this case, . Formally, one can thus write that the sensitivity is equal to
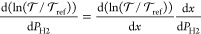
5

To assess the second term, , we reconsider the neutron reflectometry results of Figure 4a. In this figure, the slope equals , and it can be seen that this slope is ∼50% larger for Ta_0.88_Ru_0.12_ than for pure Ta. On the basis of these results, one would thus expect a ∼50% higher sensitivity for this material as compared to tantalum. Despite the fact that the sensitivity is slightly increased at higher pressures, a 50% increase is not observed in Figure 9. This thus implies that the other contribution to the sensitivity, , should thus be reduced with Ru substitution.

**Figure 10:**
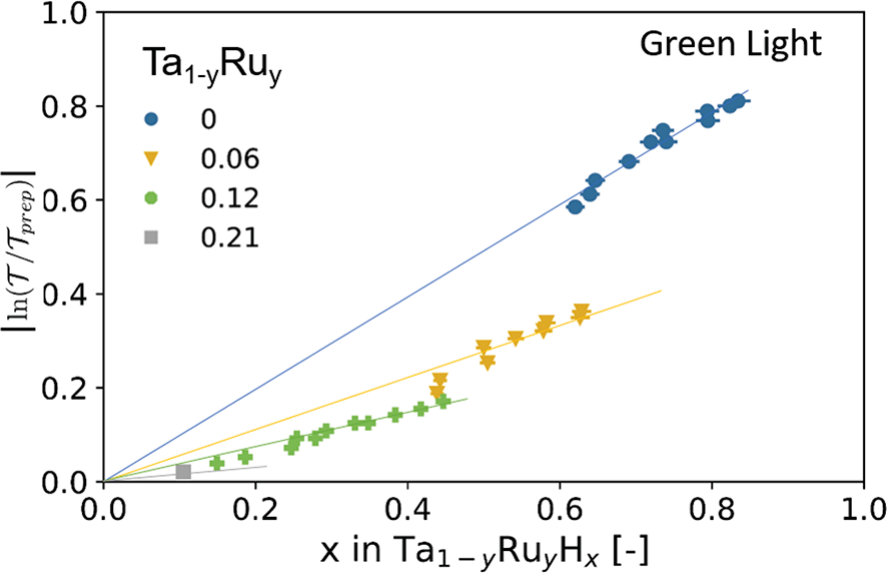
Relation between the hydrogenation and the absolute changes of the green-light optical transmission relative to the as-prepared state  of the Ta_1–*y*_Ru_*y*_ layer. The lines serve as guides to the eyes.

To investigate the evolution of the  term with Ru substitution, we plot in Figure 10 the changes of the green-light optical transmission  as a function of the hydrogen concentration in the Ta_1–*y*_Ru_*y*_H_*x*_ layer. To do this, we match for every pressure the hydrogen concentration determined by neutron reflectometry in Figure 4a with the corresponding value for  obtained from Figure 6. What we find is that there is a linear scaling between the amount of hydrogen absorbed by the sensing layer and the optical contrast for all Ta_1–*y*_Ru_*y*_H_*x*_ sensing layers. While this has been reported before for other metal hydrides^17,20,22,50^ including Ta at elevated temperatures,^23^ such a linear scaling is not trivial at all. Indeed, one would expect a linear scaling in the case of a two-phase system with a total hydrogen content change induced by varying phase fractions, but here a solid solution of hydrogen and Ta_1–*y*_Ru_*y*_H_*x*_ is formed.^9^ The fact that a linear scaling is still observed implies that the effect on the optical properties of absorbing hydrogen is independent of the hydrogen concentration within the solid solution and only depends on the composition of the host Ta_1–*y*_Ru_*y*_ lattice.

Most importantly, we observe that the slope  in Figure 10 decreases substantially with increasing Ru concentration, thus having a negative impact on the sensitivity of the hydrogen-sensing material. Indeed, we find that  decreases from  ≈ 1 for Ta to  ≈ 0.4 for Ta_0.88_Ru_0.12_. This decrease completely offsets the potential sensitivity gain from the increased value for  for this composition.

Ideally, for a hydrogen-sensing material one wants  to be as large as possible because differently from  it has no negative side effects such as potentially longer response times (due to more hydrogen that needs to be dissociated at the surface) or a potentially reduced mechanical stability owing to a larger volumetric expansion (although we note that with Ta-based compounds this has not been an issue). Nevertheless, the fact that the optical contrast generation  can be tuned by alloying Ta also provides positive prospects: it is a subject of further study to find elemental substitutions that can increase  and boost the sensitivity of the sensor without compromising any other sensing characteristic.

### 3.4 Comparison with Other Sensing Materials

Compared to Pd alloys, Hf- and Ta-based sensing materials feature a series of advantages: (i) they have a sensing range that extends to much lower hydrogen concentrations and also covers a much wider range of at least 7 orders of magnitude, (ii) the relative sensitivity is almost constant across the entire sensing range, and (iii) these materials do not feature any hysteresis, even for thin films. In contrast, Pd and its alloys plastically deform and therefore have a hysteretic response to hydrogen when structured as a thin film. While for nanoparticles the hysteresis can be suppressed by sufficiently large concentrations of alloyants such as Au and Ag, this leads to a strong reduction of the optical contrast.^14−21^ It is a major benefit of Ta-based materials that alloying does not directly reduce the optical contrast and thus the sensitivity of the material. A disadvantage of Hf- and Ta-based sensing materials is that these materials always need to be combined with a suitable capping layer to promote hydrogen dissociation to enable a fast response, as well as that a capping layer is needed to prevent oxidation.^26^

Among the Hf- and Ta-based sensing materials, Ta-based materials have the particular advantage that they can be used at room temperature and offer, in combination with a suitable capping layer, a fast response to changing hydrogen concentrations.^24^ While Hf features a constant and large sensitivity over at least 6 orders of magnitude in pressure, at room temperature this sensing range is situated at too low concentrations for most practical purposes. Therefore, Hf can only be used at elevated temperatures (*T* ≳ 90 °C).^22,23^ Among the identified Ta-based sensing materials, Ta_1–*y*_Ru_*y*_ offers more possibilities to tune the sensing characteristics due to the larger solid solution range of Ru in, Ta which is, for instance, limited to *y* ≈ 0.12 for Ta_1–*y*_Pd_*y*_.^24^

## 4 Conclusion

In conclusion, we have shown how alloying Ta with the smaller element Ru significantly reduces the size of the unit cell and how it can be used to tune the hydrogen-sensing properties. At the same time, the crystal structure of Ta is maintained, and Ta_1–*y*_Ru_*y*_ thin films show that even for *y* = 0.3 a solid solution of Ru and Ta is formed in the body-centered cubic phase of tantalum. When exposed to hydrogen, in situ X-ray diffraction measurements show that for all Ta_1–*y*_Ru_*y*_ compounds the unit cell expands gradually and a solid solution of hydrogen in Ta_1–*y*_Ru_*y*_H_*x*_ is formed. As there is no (first-order) phase transition or plastic deformation observed, this will enable a hysteresis-free response of the hydrogen-sensing material. Moreover, detailed in situ X-ray and neutron reflectometry measurements show that Ru doping is effective in reducing the hydrogen content and the volumetric expansion of the material, which improves the cyclability of the material. Optical measurements confirm that the smaller unit cell of the Ta_1–*y*_Ru_*y*_ alloys shifts the sensing range to higher pressures, that the sensing response is free of any hysteresis for all compounds considered, and that the reduced amount of hydrogen absorbed reduces the absolute magnitude of the optical changes until for *y* = 0.3 no optical response can be discerned. However, for *y* ≲ 0.12, the sensitivity, being the slope of the changes in optical transmission with changing hydrogen pressure/concentration, is only affected to a limited extent and in some cases even improved. As such, the doping with ruthenium is successful in reducing the hydrogen absorption, reducing the volumetric expansion, and shifting the sensing range to higher pressures/concentrations while maintaining the hysteresis-free response and high sensitivity of tantalum. In a more general perspective, these results illustrate that one can rationally tune the properties of metal hydride optical hydrogen-sensing layers by appropriate doping.

## Supporting Information Available

Spectrum of the LED lights used for the optical measurements, spectral sensitivity of the three color channels of the camera used for the optical measurements, X-ray reflectometry (XRR) measurements of the as-prepared films, table with fitted layer thickness, density, and roughness σ of the as-prepared films as obtained by XRR measurements, X-ray diffraction (XRD) measurements of the films after exposure to hydrogen, scattering length density (SLD) profiles of the in situ XRR results, in situ NR results, response time measurements, optical stability measurements upon repeated cycling, and additional van’t Hoff fits (PDF)
