# Supplementary material for: Tuning the Properties of Thin-Film TaRu for Hydrogen-Sensing Applications
Source: ACS Appl Mater Interfaces. 2023 Feb 3;15(6):8033–45. doi: 10.1021/acsami.2c20112 (PMC9940109; doi:10.1021/acsami.2c20112)
Supplement: Supplementary file 1 — am2c20112_si_001.pdf [file am2c20112_si_001.pdf]

Supporting Information
Tuning the properties of thin film TaRu for hydrogen sensing applications

Lars J. Bannenberg,[*,†] Herman Schreuders,[†] Nathan van Beugen,[†] Christy Kinane,[‡] Stephen Hall,[‡] and Bernard Dam[†]

†*Faculty of Applied Sciences, Delft University of Technology, Mekelweg 15, 2629 JB Delft, The Netherlands*

‡*ISIS neutron source, Rutherford Appleton Laboratory, STFC, UKRI, OX11 0QX Didcot, United Kingdom*

E-mail: l.j.bannenberg@tudelft.nl

1 Response times

Besides no hysteresis, a large sensing range with a sizable sensitivity, a fast and stable response are two other key requirements for optical hydrogen sensing materials. As response times are especially important for safety-applications, we investigate the response times close to the explosive limit of 4% in Air (under atmospheric conditions). In Figs. S9(a-c) we depict the normalized responses of the thin film to a series of increasing pressure steps and in Fig. S9(d-f) we display the corresponding pressure dependence of the response times, defined as the time to reach 90% of the total signal in Fig. S9(a-c). The room temperature response time of all the $Ta_{1-y}Ru_y$ based thin films shown here is below 1 s for $P_{H2} = 10^{+3} < P_{H2} < 10^{+5}$, i.e. a hydrogen concentration range of 1 to 100% under ambient conditions, while

Table S1: Sputter conditions for the samples used in the study. Each sample consists of about 4 nm of Ti (nominal), produced by 60 s sputtering of Ti at 150 W, a 40 nm (nominal) $Ta_{1-y}Ru_y$ sensing layer for which the conditions are tabulated below, and a 10 nm (nominal) $Pd_{0.6}Au_{0.4}$ layer produced by co-sputtering Pd (27.5 W) and Au (17 W) for 60 s.

$Ta_{1-y}Ru_y$	Power Ta [W]	Power Ru [W]	Time [s]
0	100	-	271
0.03	200	6	133
0.06	135	8	191
0.09	100	9	252
0.12	100	12.4	245
0.15	100	16	239
0.21	100	23	228
0.3	100	39	205

response times below 10 s are obtained for $P_{H2} = 10^{+2} < P_{H2} < 10^{+3}$. We wish to emphasize that these responses can be further accelerated by the application of a PTFE layer, which has shown to reduce the response times by a factor of 5-15.[1–3] Such fast responses are likely related to the diffusion in the $Ta_{1-y}Ru_y$ sensing layers, which is well-know to be high in bcc metal hydrides such as Ta.[4] Indeed, for other Ta-based thin films it has been shown that the response time is merely limited by the amount of hydrogen that is dissociated at the surface of the capping layer and not by the hydrogen diffusion through the sensing or capping layer.[3] This implies that even shorter response times can be realized by limiting the thickness of the $Ta_{1-y}Ru_y$ sensing layers as this reduces the total amount of hydrogen that needs to be dissociated at the surface.

2 Stability

To investigate the stability of the sensor response, we exposed the thin films to over 1000 cycles of hydrogen between $P_{H2} = 1.0$ and $4.0\ 10^{+3}$ Pa at $T = 28$ °C (only 70 cycles displayed). Figure S10(a-c) shows that the cycles are identical to each other, even after exposure to over 1000 cycles of hydrogen. Figure S10(d-f) further underscores the excellent stability and reproducibility of the optical response by showing three individual cycles selected at random

Table S2: Fitted layer thickness, density and roughness σ of the as-prepared Ta thin films with a Ti adhesion layer capped with the various compositions indicated. The fits are reported in Fig. S1. The samples with and without a PTFE layer were produced during the same deposition and have identical composition and layer thicknesses. The density of the fused quartz substrate was fixed to the literature value of 26.5 FU/nm^3 and the roughness was fitted to $\sigma = 0.3 \pm 0.1$ nm for all samples.

$Ta_{1-y}Ru_y$	0	0.03	0.06	0.09	0.12	0.15	0.2	0.3
Cap Thick [A]	97.2	98.2	96.9	95.7	95.7	99.3	96.96	98.5
Cap Dens [FU/A^3]	0.0628	0.064	0.0611	0.0613	0.0617	0.0619	0.0645	0.0605
Cap Sigma [A]	10.7	8.64	8.9	8.4	11	10.1	8.94	9.8
Mid Thick [A]	415.9	376.8	399.7	411.9	407.2	406.7	405.3	408.4
Mid Dens [FU/A^3]	0.0516	0.0527	0.0506	0.0514	0.0525	0.0532	0.0565	0.535
Mid Sigma [A]	6.1	2	4.1	3.7	7	5.7	4.6	6.1
Adhesion Thick [A]	39.9	38.7	39	37.9	39.7	37.5	37.5	37.5
Adhesion Dens [FU/A^3]	0.0569	0.0569	0.0569	0.0569	0.0569	0.0569	0.0569	0.0569
Adhesion Sigma [A]					7			6
Sub. Dens [FU/A^3]	0.0265	0.0265	0.0265	0.0265	0.0265	0.0265	0.0265	0.0265
Sub Sigma [A]	8	5	5	4	7	8	6	8

for which a very close correspondence is observed.

References

(1) Ngene, P.; Westerwaal, R. J.; Sachdeva, S.; Haije, W.; de Smet, L. C.; Dam, B. Polymer-Induced Surface Modifications of Pd-based Thin Films Leading to Improved Kinetics in Hydrogen Sensing and Energy Storage Applications. *Angewandte Chemie International Edition* **2014**, *53*, 12081–12085.

(2) Nugroho, F. A. A.; Darmadi, I.; Cusinato, L.; Susarrey-Arce, A.; Schreuders, H.; Bannenberg, L. J.; Bastos da Silva Fanta, A.; Kadkhodazadeh, S.; Wagner, J. B.; Antosiewicz, T. J.; Hellman, A.; Zhdanov, V. P.; Dam, B.; Langhammer, C. Metal-Polymer Hybrid Nanomaterials for Plasmonic Ultrafast Hydrogen Detection. *Nature Materials* **2019**, *18*, 489–495.

(3) Bannenberg, L. J.; Boshuizen, B.; Ardy Nugroho, F. A.; Schreuders, H. Hydrogenation

kinetics of metal hydride catalytic layers. *ACS applied materials & interfaces* **2021**, *13*, 52530–52541.

(4) Fukai, Y. *The Metal-Hydrogen System: Basic Bulk Properties*; Springer Science & Business Media, 2006; Vol. 21.

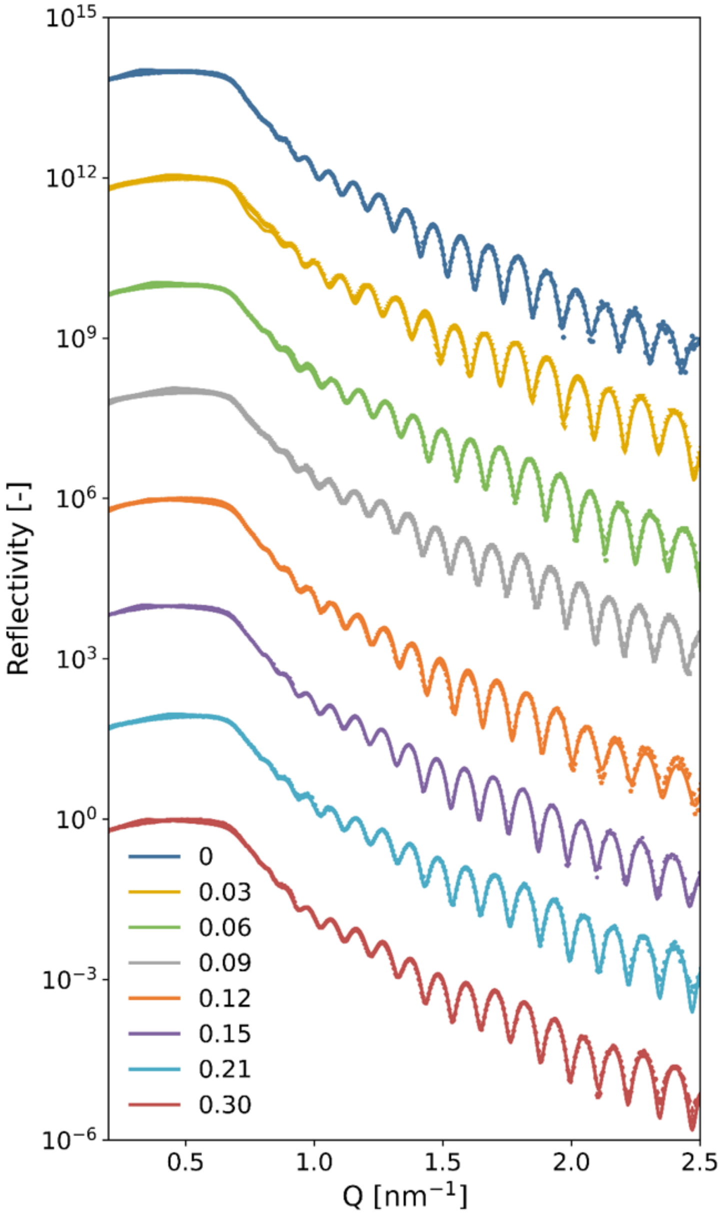

Figure S1: X-Ray Reflectometry (XRR) measurements of the as-prepared 40 nm $Ta_{1-y}Ru_y$ thin films with a 4 nm Ti adhesion layer and capped with a 10 nm $Pd_{0.6}Au_{0.35}Cu_0.05$ layer. The continuous lines represent fits of a model to the data on the basis of which estimates for the density and layer thickness are obtained that are tabulated in Table S2.

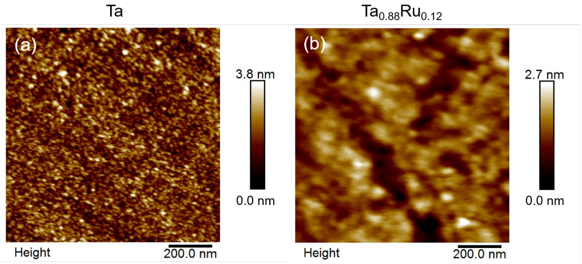

Figure S2: AFM images of the 40 nm $Ta_{1-y}Ru_y$ thin films with a 4 nm Ti adhesion layer and capped with a 10 nm $Pd_{0.6}Au_{0.35}Cu_0.05$ layer after exposure of the thin films to hydrogen and measured in air for (a) $y = 0$ and (b) $y = 0.12$. The root-mean-square roughnesses are 1 nm.

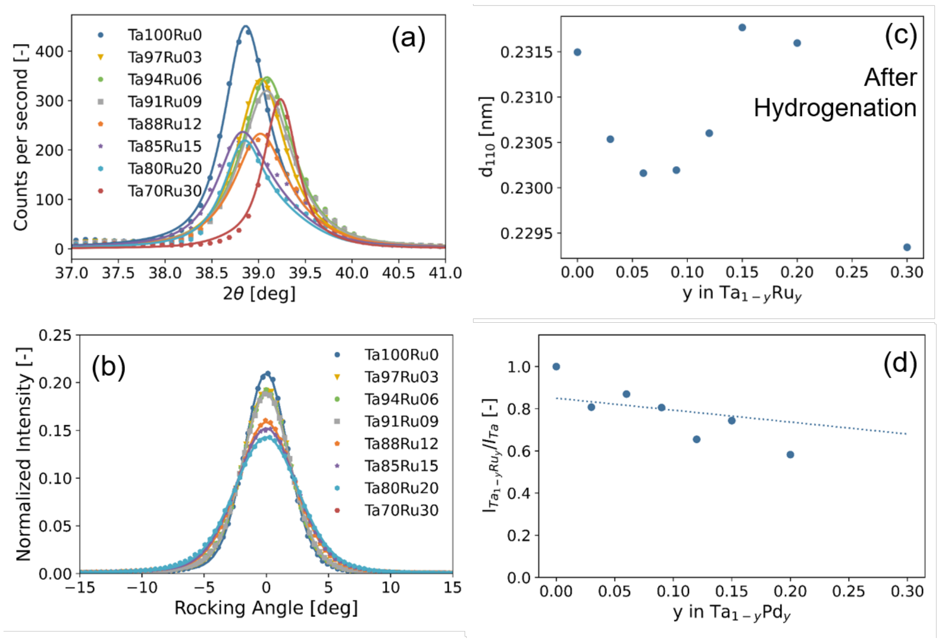

Figure S3: Ex-situ X-ray Diffraction (XRD) results of the 40 nm $Ta_{1-y}Ru_y$ thin films with a 4 nm Ti adhesion layer and capped with a 10 nm $Pd_{0.6}Au_{0.35}Cu_0$.05 layer after exposure of the thin films to hydrogen and measured in air. (a) Diffraction patterns (Cu-Kα, $\lambda = 0.1542$ nm) of the $Ta_{1-y}Ru_y$ thin films. The continuous lines represent fits of two pseudo-Voigt functions to the experimental data accounting for the bcc $\langle 110 \rangle$ $Ta_{1-y}Ru_y$ and fcc $\langle 111 \rangle$ $Pd_{0.6}Au_{0.35}Cu_0$.05 peaks. (b) Rocking curves of the $Ta_{1-y}Ru_y$ thin films around the bcc $Ta_{1-y}Ru_y$ $\langle 110 \rangle$ peak. (c) Ru doping dependence of the d_{110}-spacing in $Ta_{1-y}Ru_y$. (d) Ru concentration dependence of the total intensity of the $\langle 110 \rangle$ diffraction peak in $Ta_{1-y}Ru_y$ in which the effect of both the changing amplitude and width are incorporated. It is computed by multiplying the integrated intensity of the fitted $\langle 110 \rangle$ $Ta_{1-y}Ru_y$ peak by the FWHM of the rocking curve of (b). The intensity is subsequently scaled to the intensity of the Ta sample. The dashed line indicates the theoretically expected Ru-concentration dependence of the intensity according to eq. 2. The d-spacing and intensity for $y \gtrsim 0.15$ likely deviate from the expected relations as because of too small hydrogenation to induce reorganization of atoms (the d-spacing is for $y \gtrsim 0.15$ similar to the values of the as-prepared samples, while for $y \lesssim 0.15$ the d-spacing is reduced substantially).

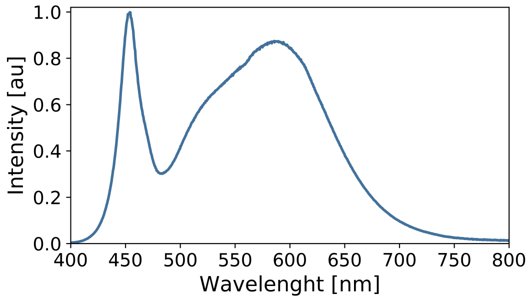

Figure S4: Spectrum of the Philips MR16 MASTER LEDs (10/50 W) with a color temperature of 4,000 K used for the white-light optical transmission (hydrogenography) measurements.

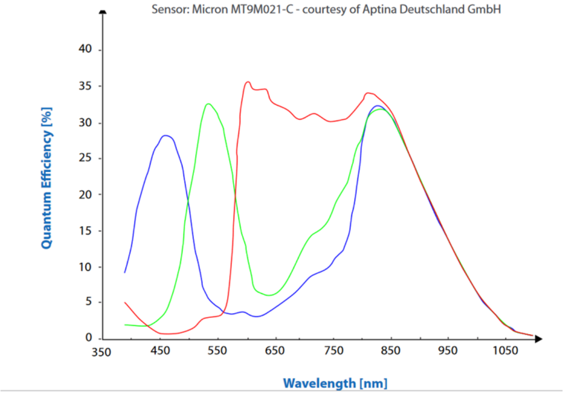

Figure S5: Spectral sensitivity of the three color channels of the Imaging Source DFK 23UM021 1/3 " Aptina CMOS MT9M021 color camera.

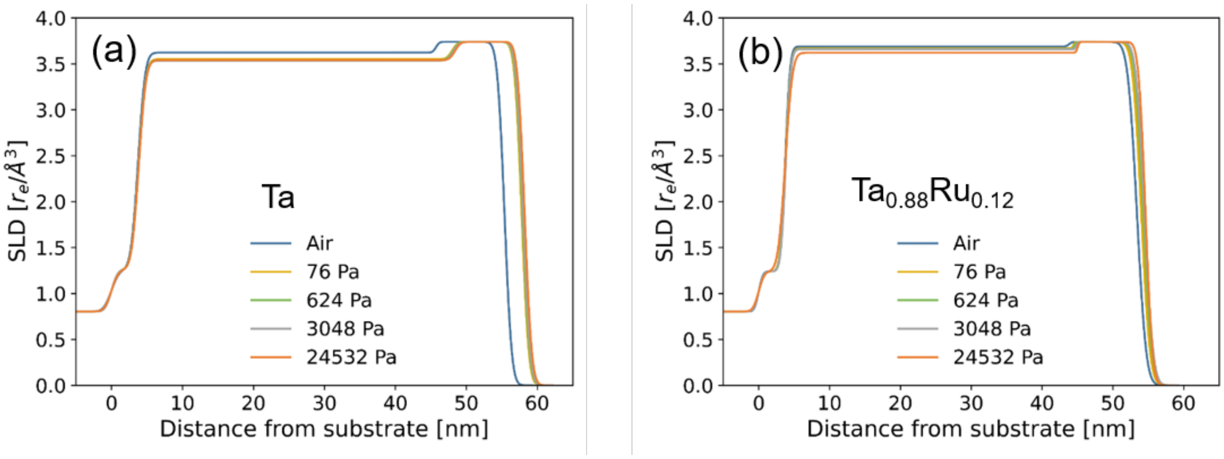

Figure S6: Scattering length density (SLD) profiles of the in-situ XRR results of the 40 nm $Ta_{1-y}Ru_y$ thin films with a 4 nm Ti adhesion layer and capped with a 10 nm $Pd_{0.6}Au_{0.35}Cu_{0.05}$ at $T = 28$ °C displayed in Fig. 3.

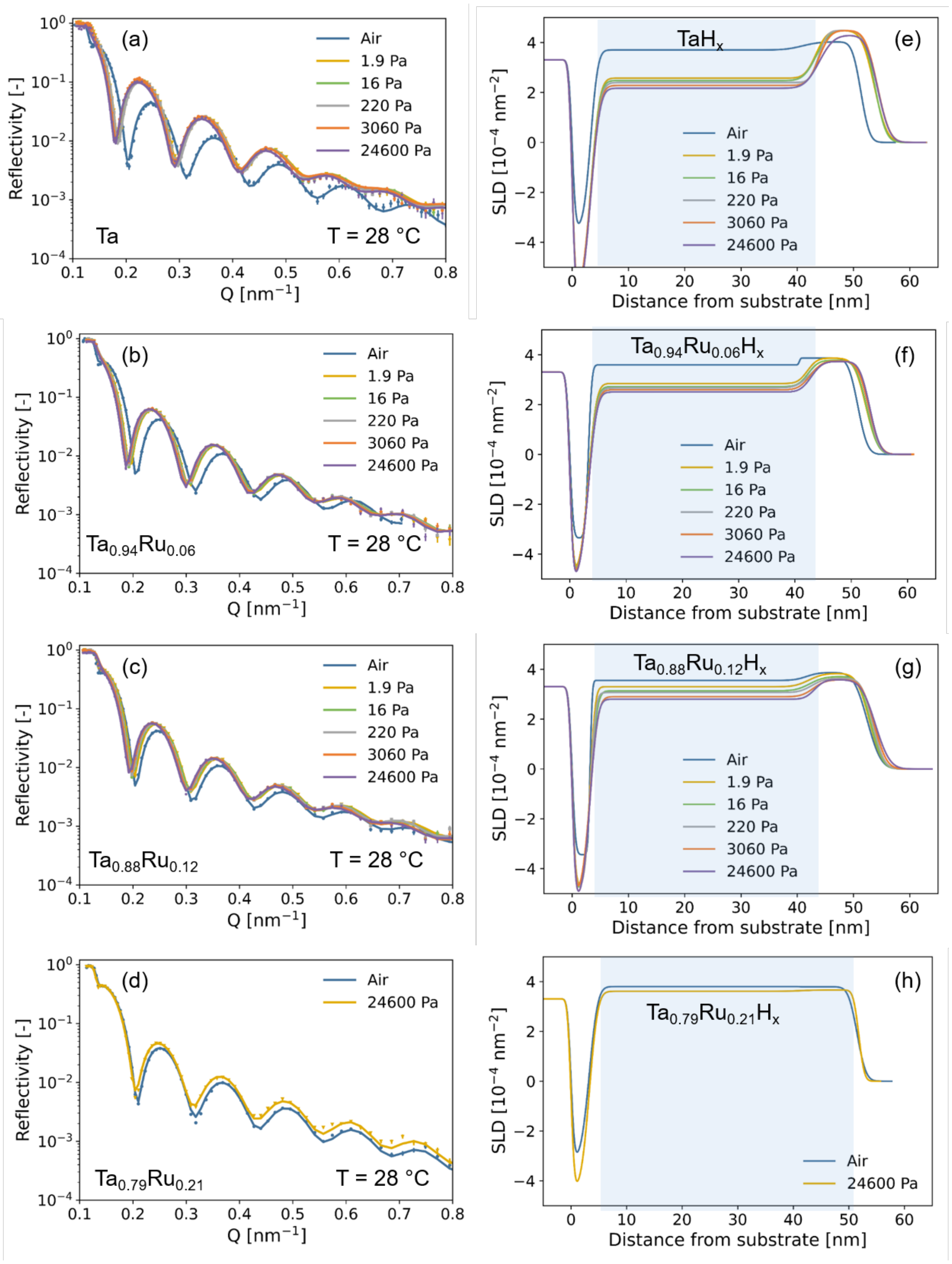

Figure S7: In-situ NR results of the 40 nm $Ta_{1-y}Ru_y$ thin films with a 4 nm Ti adhesion layer and capped with a 10 nm $Pd_{0.6}Au_{0.35}Cu_{0.05}$ at $T = 22$ °C. (a-d) Reflectograms of the $Ta_{1-y}Ru_y$ thin films measured for the hydrogen pressures indicated in the legend and for increasing pressure steps. The continuous lines represent fits of a model to the data on the basis of which estimates for the scattering length density and layer thickness are obtained (see Fig. 4). (e-h) Scattering length density (SLD) profiles.

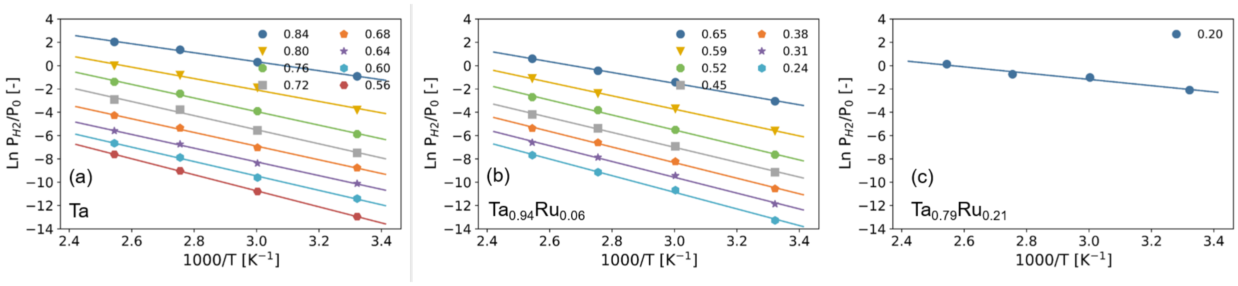

Figure S8: Fits of Van 't Hoff's law (eq. 3) to the experimental data for the samples indicated as based on the temperature-dependent optical transmission data of Figure 7. The optical transmission is then converted to the hydrogen-to-metal ratio as provided in the figure legend using the scaling obtained in Figure 10. The enthalpy and entropy of the hydrogenation reaction obtained from the fits are reported in Figures 8(b) and (c), respectively.

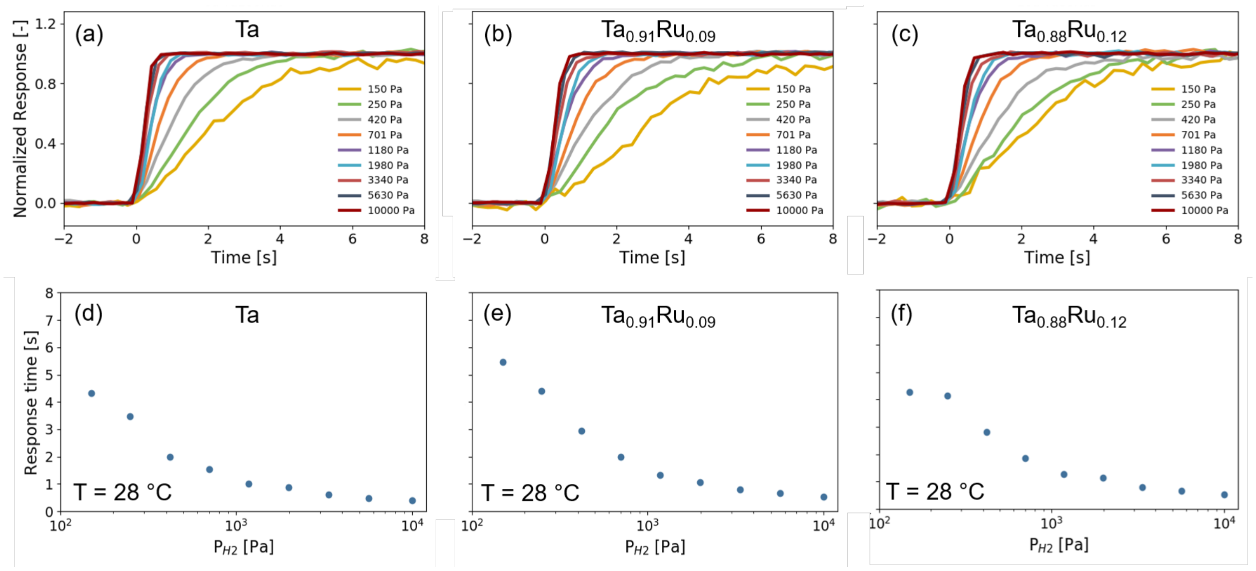

Figure S9: Absorption kinetics of 40 nm $Ta_{1-y}Ru_y$ thin films with a 4 nm Ti adhesion layer capped with a 10 nm $Pd_{0.60}Au_{0.35}Cu_{0.05}$ layer at $T = 28$ °C. (a-c) Normalized responses of the thin films to a series of pressure steps between $P_{H2} = 0.5\ 10^2$ Pa and the partial hydrogen pressure indicated. (d-f) Hydrogen pressure dependence of the response time of the thin film. The response time is defined as the time to reach 90% of the total signal.

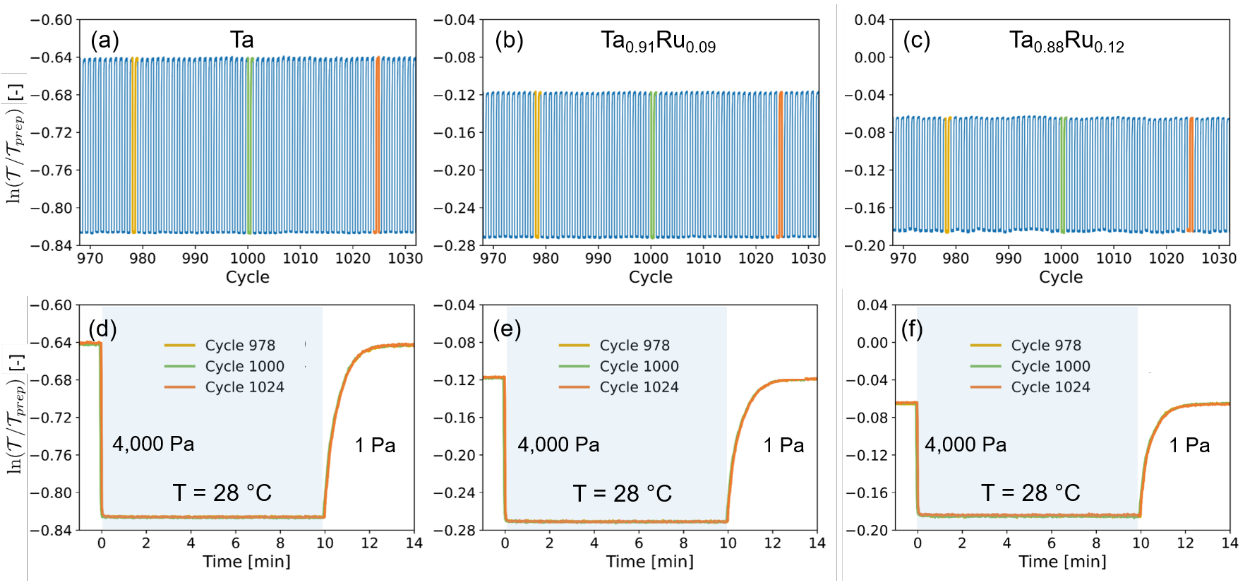

Figure S10: Stability of a 40 nm $Ta_{1-y}Ru_y$ thin film with a 4 nm Ti adhesion layer capped with a 10 nm $Pd_{0.60}Au_{0.35}Cu_{0.05}$ layer. The green light optical transmission is measured relative to the optical transmission of the as-prepared state ($\mathcal{T}_{prep}$). (a-c) Optical response of the films to 70 (out of 1000) selected cycles in which the pressure was varied between partial hydrogenation (P_{H2} = 4.0 10^{+3} Pa) and partial dehydrogenation (P_{H2} = 1 Pa) at T = 28 °C. (d-f) The identical behavior of three individual hydrogenation cycles selected at random from (a-c). The slow desorption mainly originates from the slow removal of hydrogen from the measurement cell.
